# Dataset on environmental, social and governance information firms and their merger and acquisitions activities

**DOI:** 10.1016/j.dib.2023.109457

**Published:** 2023-07-28

**Authors:** Andreas Dimmelmeier

**Affiliations:** Institut für Statistik, Ludwig-Maximilians-Universität München, Ludwigstr. 33, 80539 München

**Keywords:** ESG, ESG information firms, Sustainable finance, Sustainable investment, Political economy, Data Intermediaries, Network analysis

## Abstract

The increasing salience of sustainability concerns in financial markets has led to growing demand by financial institutions, regulatory authorities and other stakeholders for information on the Environmental, Social and Governance (ESG) credentials of companies. To address these demands, private firms providing ESG data and related products have expanded in size and in number. Reflecting the increased importance of ESG data, academics, sustainable finance practitioners and financial regulators have surveyed the emerging industry of these ESG information firms. However, to date no structured dataset on the characteristics of ESG information firms is available. The ESG Information Firms Dataset (ESGFiDa) presented in this article is a first step to address this gap. ESGFiDa consolidates the findings from the academic and gray literature on the ESG information industry and augments these with additional data gathered through desk research. Based on this multi-stage data collection process, the dataset provides 1) a meta-analysis of existing accounts of ESG information firms from a variety of sources and 2) consolidated and augmented data about 128 ESG information firms. The meta-analysis part of the dataset contains amongst others information on different estimates regarding the size of the ESG information industry that have been proposed in the literature. The second part of ESGFiDa provides data on 128 ESG information firms that have been identified from the literature and consists of five variables (Name, Type, Country, Continent, Year of incorporation, and Headquarters). In addition, mergers and acquisitions among the 128 ESG information firms are recorded in a dated and geolocated edgelist with 65 entries. These relational data can be used for network analysis. In summary, ESGFiDa offers a first systematized description of the dynamics of the emerging ESG information industry. As such, it can be of interest to students of sustainable finance from fields including finance and economics, political science, organization studies, sustainability studies and financial geography. Moreover, practitioners from financial institutions interested in the backgrounds and differences of ESG information firms as well as policymakers and financial regulators tasked with overseeing and regulating the emerging ESG information industry might find the presented dataset useful.

**Specifications Table**

**Table:** 

Subject	Financial Markets and Institutions
Specific subject area	ESG information firms, i.e. commercial actors intermediating between corporates and financial institutions by compiling and analyzing ESG data
Data format	Raw, Analyzed, Cleaned
Type of data	Table, Image, Chart, Graph, Figure
Data collection	Data were collected through desk research. The first part of this research consisted of screening, aggregating and systematizing information from the academic and gray literature. Subsequently, information from these sources about ESG information firms was augmented by consulting specialist news and data providers.
Data source location	Data sources include academic, regulatory and industry publications, specialist financial press (e.g. Bloomberg, Responsible Investor), company registry websites, web-archives (e.g. Wayback Machine) and company presentations on LinkedIn and Wikipedia
Data accessibility	Repository name: Open Science Foundation (OSF) Data identification number: 10.17605/OSF.IO/B7ZGD Direct URL to data: https://osf.io/b7zgd/

## Value of the Data

• This dataset aggregates and augments disjoined information about ESG information firms. Researchers and practitioners can use the data to complement historical accounts and hypotheses about the evolution of the ESG information industry, which so far have mostly relied on qualitative data and studies of small numbers of ESG information firms.
• The data can be linked with other qualitative and quantitative data relating to, for instance, changes in the methodologies of ESG information firms, the enactment of sustainable finance regulations across different jurisdictions and the adoption of sustainable finance practices by investors. By connecting existing datasets and observations on sustainable finance with the present dataset on the dynamics of ESG information firms, novel hypotheses regarding the emergence and governance of sustainable finance can be developed and tested.
• As the ESG information industry appears to be a highly dynamic emerging sector, the data structure is also a potential starting point for future academic data collection or fact-finding surveys from policymakers and financial regulators.
• Researchers interested in more in-depth qualitative studies of ESG information firms might use the dataset for their case selection.

## 1 Data Description

The data in ESGFiDa is stored in an .xlsx file across 6 sheets. Sheet 1 contains the general description of the dataset and the variables, while the data themselves are stored in sheets 2 to 6. Excluding, excluding id values, two double entries of variables before and after data cleaning (Literature_ESG_estimates and Name_orga) and two columns containing explanatory comments, ESGFiDa consists of 21 variables. 13 of these variables belong to the meta-analysis of the literature part and 8 to the ESG information firms part of the dataset. Moreover, the dataset contains an edgelist that provides information about the merger and acquisition (M&A) relations among ESG information firms (sheet 6). The edgelist adds 5 additional features to the ESG information firm part. Table 1 provides a detailed overview of the variable names, descriptions, types and values.

**Table 1:** Detailed description of the variables of ESGFiDa [1].

Detailed overview of individual sheets and variables				
Variable Name	Variable Description	Variable Type	Description of observations and possible values	Part of ESGFiDa
**Sheet 2 Literature (*n* = 15–25 dependent on variable name)**				
Literature_ESG_general	Articles and reports providing an overview of the ESG information industry	character	Author Names Year of Publication: Title	Meta-analysis
Literature_ESG_estimates	Articles and reports providing a numerical estimate of the size of the ESG industry	character	Author Names Year of Publication: Title	Meta-analysis
Literature_ESG_estimates_clean	Equivalent to Literature_ESG_estimates minus one report that could not be located	character	Author Names Year of Publication: Title	Meta-analysis
Literature_sources_dataset	Articles and reports with lists or in-text mentions of ESG information firms	character	Author Names Year of Publication: Title	Meta-analysis
**Sheet 3 Estimates ESG entities (*n* = 27)**				
Name_Article_or_Report	Title of article or report	character	Title: Subtitle, NA if published on a website without title	Meta-analysis
Authors_Article_or_Report	Full name of mentioned authors	character	Forename, Surname, NA if published by an organization and no names are provided	Meta-analysis
Publishers	Organization or outlet (e.g. book, journal) responsible for publishing article or report	character	Name of publishing organization or outlet	Meta-analysis
Link	Hyperlink to article or report	hyperlink	hyperlink	Meta-analysis
Number_ESG_entities	Number of ESG entities (e.g. agencies, standards, indexes) mentioned in the article or report OR sum of entries in case a list of ESG entities is provided	numerical	range: 7 - 652	Meta-analysis
Terminology_ESG_entities	Wording used to refer to the assessed ESG entities (e.g. ESG rating agencies)	character	exact quote preceding numerical information OR title of list	Meta-analysis
Definition_ESG entities_provided (Y/N)	Assessment whether the report or article provides an explicit definition of the ESG entities it deals with	binary	Yes, No	Meta-analysis
Source_Type	Classification of the background of the source	nominal	Academic publication = peer reviewed journal article, edited volume chapter, monograph or pre-print; Industry report = consultancy study or presentation, trade association publication or website; regulatory report = study written or commissioned by public national or supranational (e.g. EU) financial authority	Meta-analysis
Publication_Year	Year of publication of article or report as indicated in the document	categorical (date)	range: 2005–2023	Meta-analysis
Data_collection_method	Description of how the number of ESG entities was assessed. If the article or report has a methodology section, the description is directly taken from there	character	Desk research, surveys, existing datasets, interviews; “not disclosed” if no information is provided	Meta-analysis
Comments	Researcher comments on data entry	character	Geographical limitations of articles, qualifications of estimates of ESG entities (e.g. “more than”, “between”)	Meta-analysis
**Sheet 4 Organisations pre-filtered (*n* = 148)**				
Name_orga	Name of the organization	character	Full name or official acronym	Aggregated ESG information firms dataset
Included (Y/N)	Assessment of whether the organization is an ESG information firm. Justifications for exclusion are provided in the variable Exclusion_justification.	binary	Yes, No	Aggregated ESG information firms dataset
Exclusion_justification	If Included (Y/N) has the value 'N', this variable justifies the assignment of the 'N' value	nominal	non ESG company = company does not fit the definition of ESG firm provided in section EXPERIMENTAL DESIGN, MATERIALS AND METHODS, no data found = no background information on the organization could be found through web searches, company websites, web archives and company registries, subdivision or product of other company = the organization listed has been attributed to another ESG firm, misspelling = the organization is equivalent to another organization already in the dataset but is misspelt	Aggregated ESG information firms dataset
Comments	Researcher comments on data entry	character	additional information on the dissolution of ESG firms that have not merged	Aggregated ESG information firms dataset
**Sheet 5 ESG data firms (*n* = 128)**				
id	Numerical id number for each observation	nominal	range: 1 - 128	Aggregated ESG information firms dataset
Name_ESG	Name of the organization	character	Full name or official acronym	Aggregated ESG information firms dataset
Type	Classification of the background of ESG information firm into four types	nominal	SRI = Socially Responsible Investment firm, designating “traditional” ESG research that first emerged against the background of the Responsible Investment movement. Observations in this category refer to “pure-play” ESG firms, whose core products are company scores and ratings, Model = Specialized firms that use models, (large and proprietory) datasets, artificial intelligence or other technologies to generate ESG data. Firms classified as “Model” distinguish themselves from traditional SRI firms by laying less emphasis on human ESG analysts and instead foregrounding technological systems, Finance = mainstream financial data providers that generate and aggregate business and financial metrics (e.g. credit risk, profit and loss, indexes), other = remaining observations that cannot be sorted into the other categories. Includes professional services firms and consultancies as well as conglomerates with subsidiaries across different sectors	Aggregated ESG information firms dataset
Country	Country, where the ESG information firm (on a consolidated level) is incorporated	nominal	Country names according to ISO 3166–1	Aggregated ESG information firms dataset
Continent	Continent, where the ESG information firm (on a consolidated level) is incorporated	nominal	Continent names according to Our World in Data	Aggregated ESG information firms dataset
Year_Founded	Year, in which the ESG information firm was incorporated	categorical (date)	range = 1846 - 2018	Aggregated ESG information firms dataset
Headquarters	City, where the ESG information firm's headquarters is located	nominal	City names in English	Aggregated ESG information firms dataset
**Sheet 6 M&A ESG data firms (*n* = 65)**				
Source	id (as specified on Sheet 4 id) of the acquired ESG information firm	nominal	range: 1 - 128	Aggregated ESG information firms dataset
Target	id (as specified on Sheet 4 id) of the acquiring ESG information firm	nominal	range: 1 - 128	Aggregated ESG information firms dataset
Time	Year of the merger or acquisition	categorical (date)	range = 2000 - 2022	Aggregated ESG information firms dataset
from	City, where the acquired ESG information firm's headquarters is located	nominal	City names in English	Aggregated ESG information firms dataset
to	City, where the acquiring ESG information firm's headquarters is located	nominal	City names in English	Aggregated ESG information firms dataset

Table 1 presents the variables in the order they are displayed on the different sheets of the dataset. Variables concerning the meta-analysis of the literature are stored in sheets 2 and 3, while data about ESG information firms can be found in sheets 4 to 6. As the sheets refer to different aspects and units of observations, they contain different numbers of rows. Moreover, sheets 1 and 4 provide information about the sources for data collection and the process of data-cleaning respectively (see section EXPERIMENTAL DESIGN, MATERIALS AND METHODS) and are hence not directly relevant for users. Excluding sheets 1 and 4 as well as qualitative researcher comments providing context on some observations and id values, the database contains a total of 1363 observations.^1^

### 1.1 Meta-analysis of the literature

With regards to the review of the existing literature, the variables in the dataset record the type of publication (i.e. academic publication, industry report, regulatory report), the estimated size of the ESG information industry, that is the number of ESG entities that a given publication finds, as well as the year of publication and the terminology used across articles or reports. With regards to the type of publication, 14 observations are academic publications published in peer reviewed journals, edited volumes or monographs, 10 are industry and consultancy reports and 3 are regulator reports. The time of publication ranges from 2005 to 2023 but more than half of the articles or reports (15) were published between 2015 and 2020.

**Fig. 1:**
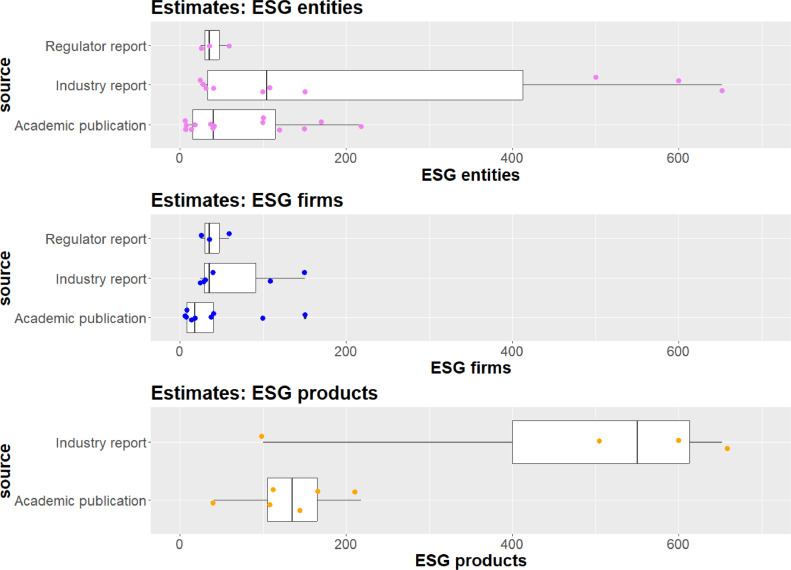
Distribution of estimates on the size of the ESG industry by publication type and terminology. The size of the boxplots shows the interquartile range - 25th to 75th percentile - with the median value as a black line.

The number of reported ESG entities across the 27 articles ranges from 6 to 652 with a mean of 125 (rounded) and a median of 41. As can be observed in the first panel of Fig. 1, there is a substantial variation in the ranges depending on the source type of the estimates. Academic publications and regulatory reports in particular find in their majority that the number of ESG entities is in the lower two-digit range with median values of 41 (rounded) and 35 respectively, while the same statistic is 104 for industry reports.

In addition, to differentiating the estimates on the size of the ESG information industry by the type of publication, the dataset contains a manually executed segmentation that takes the terminology used in the articles or reports into account. This segmentation is created by separating terms like “ESG rating agencies”, “ESG data vendors” or “sustainability analyst organisations” that refer to firms and organisations from words such as “Ratings, Rankings and Indexes”, “ESG products” or “ESG brand rankings” that refer to products. Executing this differentiation results in two subsamples for organisations (19 observations) and products (8 observations). The lower two panels of Fig. 1 respectively show the distribution of firms and products according to the different publication types.

### 1.2 ESG information firms’ data

#### 1.2.1 Attributes of ESG information firms

Sheets 5 and 6 present the consolidated dataset on ESG information firms that covers 128 observations. The data contain information about the firms' geographical location, year of incorporation, and type as well as the M&A dynamics among them. The variable “type” categorizes the backgrounds and business models of ESG information firms. The categories for the type variable are “Socially Responsible Investment (SRI)”, “Model”, “Finance” and “Other”. These categories are derived from discussions and typologies in the literature about sustainable finance and ESG in general [2], [3], [4], [5] and ESG information firms in particular [6], [7], [8]. As detailed in the variable description in Table 1, the SRI category covers “traditional” ESG research firms that first emerged against the background of the Corporate Social Responsibility and Responsible Investment movements. The Model category, meanwhile, contains specialized firms that mobilize modeling techniques, large and often proprietary datasets, artificial intelligence or other technologies to generate ESG data. ESG information firms classified as “Finance” refer to mainstream financial data and service providers that generate and aggregate business and financial metrics. Well-known examples of this type of firms are MSCI, S&P Global or Bloomberg. Finally, the “Other” category collects the remaining observations and includes professional services firms and consultancies as well as conglomerates with subsidiaries across different sectors.

**Fig. 2:**
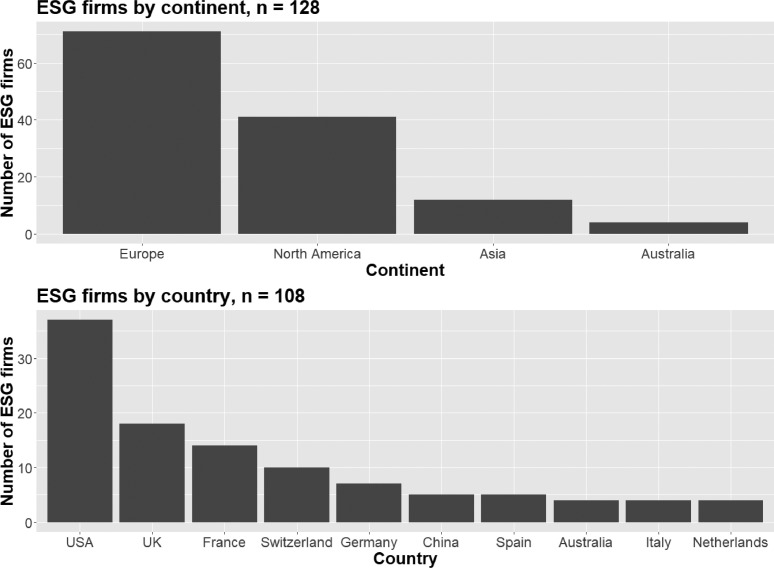
Geographical distribution of the ESG information firms contained in ESGFiDa by continent (first panel) and country of origin (second panel).

**Fig. 3:**
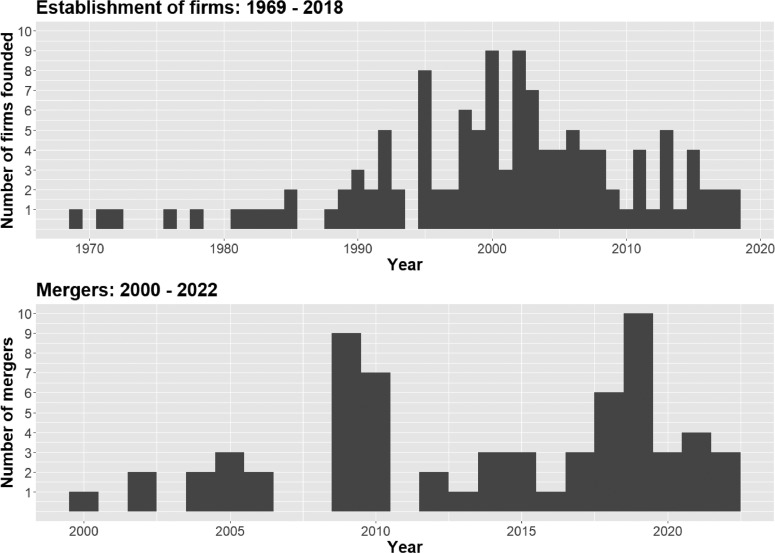
Histograms of the time-related variables in ESGFiDa. The first panel presents the founding dates of 122 of the 128 ESG information firms (6 firms established before 1969 were excluded). The second panel shows the occurrence dates of 65 Mergers & acquisition events between 2000 and 2022.

**Fig. 4:**
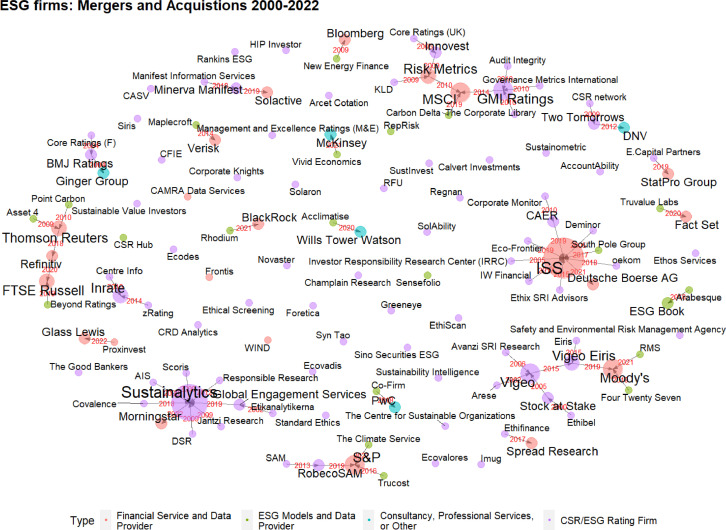
Network of M&A relations among ESG information firms. Firms’ names are labelled black, M&A dates are displayed in red. Nodes are sized by in-degree. Colours indicate the firms’ type classification.

The distributions of the categorical variables described in the previous are visually presented in Fig. 2, Fig. 3, Fig. 4. Fig. 2 plots the geographical distribution of ESG firms by continent and country. As shown in the first panel of the figure, most firms are located in Europe (71 firms or 55,5%) and North America (41 firms or 32,0%) with only 16 companies (12,5%) not coming from either region. The second panel of Fig. 2, which for reasons of space only displays the top 10 countries in terms of the presence of ESG information firms, shows that most firms are hosted by the United States (37 or 28,9%). The UK, France, and Switzerland are also well represented in the distribution with each country hosting 18, 14, and 10 ESG information firms respectively. Further disaggregation of the geographical distribution of ESG information firms at the headquarter city level can also be derived from the dataset but is for reasons of space not illustrated graphically here.

Fig. 3 shows the distribution of the observations relating to the temporal dimension of the dataset. The first panel displays the values concerning ESG data firms’ year of establishment in a histogram. The second panel shows the distribution of M&A events in the same format. For reasons of interpretability, six observations with extreme value relating to incorporations before 1969 have been dropped from the representation in panel one. The six firms founded before this cut-off date are all mainstream financial service and data providers that have only recently entered the ESG information business through M&A (on the rationale for including these firms see the definitional boundaries outlined in section EXPERIMENTAL METHODS, MATERIALS AND METHODS).

As to the year of incorporation, Fig. 3 shows that the establishment of ESG information firms started in the 1970s and 1980s. The peak of the creation of new firms was in the early 2000s. Since then the pace of founding new firms has smoothened. The second panel of Fig. 3 suggests, that M&A activities were particularly pronounced in 2019 (10 mergers), 2009 (9 mergers), 2010 (7 mergers), and 2018 (6 mergers).

#### 1.2.2 M&A network of esg information firms

The coding of M&A activities as edges among ESG information firms also allows for network analysis and network representation of the data contained in ESGFiDa. One possible visualization of these network relations is illustrated in Fig. 4. The graph depicts ESG information firms as nodes and M&A activities as directed and dated edges. In the figure, the arrows of the edges point from the acquired firm to the acquiring firm. Moreover, the year of the M&A event is shown next to the edge in red. The size of the nodes and the firms’ labels is scaled according to the number of firms they acquired or in network analysis terminology their “in-degree”. Node colours, meanwhile, reflect the categorizations from the type variable. As illustrated in Fig. 4, most ESG information firms are -SRI oriented companies (purple, 62,5%). Financial services and data providers and model-based firms respectively account for 22 (red, 17,2%) and 21 (green, 16,4%) observations. The “other” category that groups professional services firms with consultancies and multi-sector conglomerates consists of 5 observations (turquoise, 3,9%).

It should be noted that the graphically displayed node attributes in Fig. 4 were only chosen for illustrative purposes and depending on the interests of researchers, other features such as geographical information or year of incorporation could be readily included in visualizations.

## 2 Experimental Design, Materials and Methods

**Fig. 5:**
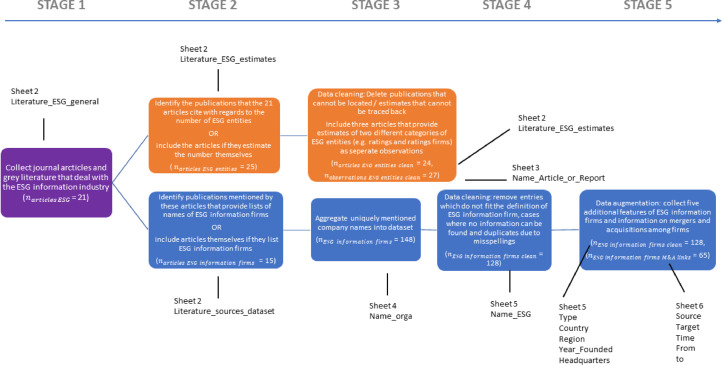
ESGFiDA data collection and data cleaning process segmented into five stages and two parts. The data collection for both parts of the dataset begins with the collection of generalist publications on the ESG information industry (purple box). Subsequently, additional publications are identified and cleaned for the meta-analysis of the literature in two additional stages (orange boxes). For the second part pertaining to the construction of a consolidated dataset of ESG information firms, the initially collected publications from stage one are first complemented by additional articles and reports (stage two). Subsequently, the names of ESG information firms are extracted from this new collection of publications (stage three). In stage four the list of names of ESG information firms is cleaned to remove inadequate entries. Stage five augments the information on the ESG information firms that have been obtained from the literature through dedicated desk research and the systematization of data points.

The dataset was developed in an iterative process that combined literature review with targeted desk research to augment findings from the literature. The stages of the research process are graphically described from left to right in Fig. 5. In addition, Fig. 5 refers back to the sheet structure of the dataset by directing the reader to the sheets and variables that are the result of a particular stage of the research process.

Starting from the left side of Fig. 5, the purple box illustrates the first stage of data collection, which consisted in compiling publications that offer an overview of the ESG information industry. In the absence of dedicated industry classifications, databases or academic research on the ESG information industry, this first stage consisted in collating articles and reports that concern themselves with the history of the industry, its consolidation and problems. To find these articles, two research strategies were applied. First, publications concerning the ESG information industry from “authoritative sources” such as regulators and standard setters like the European Securities and Markets Authority, the International Organization of Securities Commissions, the European Commission and the longstanding “Rate the Raters” project were collected [[9], [10], [11], [12]]. Second, academic publications were collected through a snowball search that started from recent historical and comparative treatments of sustainable finance in general and ESG information firms in particular [2,4,5,[13], [14], [15]]. Screening these articles for references regarding the size and delineation of the ESG information industry led to the identification of additional literature. Employing these two strategies in the first stage of data collection resulted in the identification of 21 articles and reports. The titles and authors of these publications are stored in Sheet 2 of ESGFiDA in the “Literature_ESG_general” column.

The articles and reports collected in stage one served as the basis for the subsequent data collection concerning the meta-analysis of the literature on the ESG information industry on the one hand (orange boxes in Fig. 5), and the building of a consolidated dataset on ESG information firms on the other hand (blue boxes in Fig. 5). With regards to the meta-analysis part of ESGFiDa, the publications collected in stage one were screened for information regarding the “size” of the ESG industry (proxied by the number of ESG rating agencies, ESG products or similar terminology – see also Sheet 3 ESG_entities_terminology). Wherever the mentioned estimates of the size of the ESG industry were attributed to another publication, this publication was tracked down and included in the dataset of the meta-analysis. This process led to the identification of a total of 25 publications.

**Fig. 6:**
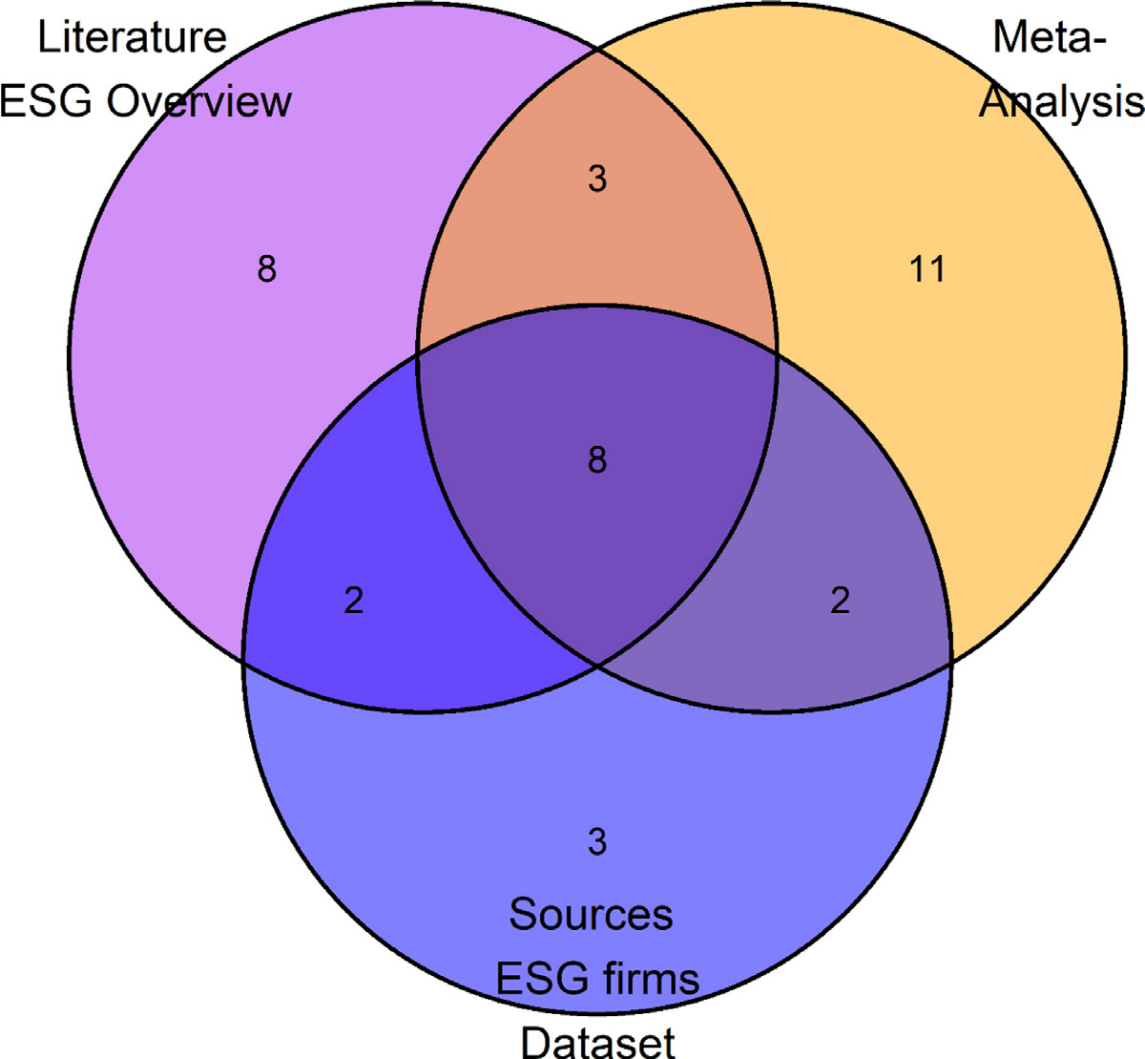
Venn diagram of the overlaps of the articles and reports used for different parts and stages of building the dataset. The purple circle refers to the literature collected in stage one (21 publications). The yellow circle refers to the literature used for the meta-analysis (24 publications). The blue circle captures the publications from whom the names of ESG information firms were extracted (15 publications).

The combination of articles and reports from stage one that themselves undertake estimates of the size of the ESG information industry with additional publications in stage two of the data collection on the meta-analysis part of ESGFiDa leads to a partial overlap of the observations included in either stage. As illustrated in the Venn diagram displayed in Fig. 6, there are 11 overlapping publications between stage one (purple circle) and stage two of the meta-analysis (yellow circle).

Following the identification of the 25 publications that provide numerical estimates of the size of the ESG information industry, stage three of the meta-analysis discarded one publication, whose location could not be tracked down. Moreover, in stage three, three publications, each of which contains two different estimates regarding different categories employed to proxy the ESG information industry (e.g. ESG ratings AND ESG rating agencies) were twice encoded in sheet 3.

To construct the second part of ESGFiDa, i.e. consolidated dataset on ESG information firms, the articles and reports collected in stage one were screened for lists and tables that provide the names and additional variables on ESG information firms. Mirroring the process applied for the meta-analysis, additional publications were brought into the dataset in the second stage of data collection if the original publications did not provide data on ESG information firms themselves but instead referenced other sources. In stage three, all the unique ESG information firms, i.e. not recording the same firm name multiple times if mentioned in different articles, that were identified through this process (*n* = 148) were stored in sheet 4 in the Name_orga column.

These 148 observations were subjected to data cleaning in stage four of the construction of the ESG information firms dataset, which resulted in a final count of 128 ESG information firms (see sheet 5 Name_ESG). The data cleaning stage consisted in screening out misspellings of the same ESG information firms across different publications (1 case), instances where subdivisions or products of an already included ESG firm were initially included as independent observations (5 cases), ESG information firms, whose existence could not be verified through additional desk research (8 cases, on the sources for desk research, see stage five below) and firms, which upon closer inspection did not classify as ESG information firms (6 cases). The justifications for excluding 20 observations from the initial collection of 148 organizations are recorded on sheet 4 in the column Exclusion_justification.

With regards to the exclusion criterion of non-correspondence to the definition of an ESG information firm, it should be noted that following Scalet and Kelly [16], I define an ESG information firm as an organization that provides and markets CSR and/or ESG data and related products. ESG data in this context refer to the sustainability performance across the E, S, and G categories of corporates and other organizations. In addition, I follow Demartini [6] and Novethic [17], who deliberately exclude NGOs, research institutes and in-house research teams of financial institutions engaged in ESG data creation and analysis from their data collection. This exclusion derives from the fact that these actors do not provide data and analyses for profit and, as such, can hardly be considered as belonging to an “industry”. To execute the network analysis of the M&A relations of the firms, I, however, include companies that acquire ESG information firms in the definition of “ESG information firm”. This definitional choice replicates the approach developed in a conference paper that presented an earlier version of the M&A data [18].

The fifth stage of the data collection process for the ESG information firms part consisted of adding additional features to the 128 observations. These features were retrieved from the previously reviewed academic, industry and regulatory publications as well as from specialist news and data providers (e.g. Bloomberg, Responsible Investor, company registry websites) company websites (including web archives and *wayback machine* for defunct or merged companies) and company online information on LinkedIn and Wikipedia. Features of individual ESG firms are recorded on sheet 5, while relational data regarding firms' mergers and acquisitions that can be used for network analyses are stored on sheet 6.

All analyses and modifications of the data that are presented in this article were carried out in the R environment. The script for reproducing these operations can be accessed through the Open Science Foundation Repository link that is attached to this publication.

## 3 Limitations

As the initial data collection stage for constructing ESGFiDa is based on the literature on sustainable finance and the ESG information industry, biases and omissions that have been observed in existing accounts likely also apply to the dataset. One noteworthy aspect is that the selection of exclusively English-language publications^2^ means that the geographical distribution of the ESG information firms might be subject to selection bias.

A second possible limitation relates to the relative coarseness of some of the variables. The encoding of M&A events between ESG information firms, for instance, does not account for firm and merger-specific idiosyncrasies. By way of illustration, some mergers result in a full integration of the acquired organization into the processes of the newly consolidated entity. In other cases, by contrast, different acquired business units retain their processes and often also their branding while being coordinated by an organizational structure such as a holding. Another issue related to coarseness could be present in the variables about ESG firms’ geographic location like the country of origin and the location of the corporate headquarters. While these variables might contain meaningful information about decision-making power for firms that display strongly hierarchical organizations, they might be less relevant for firms that afford considerable discretion to their subsidiaries.

In addition to these limitations, there are some caveats related to the data collection and classification process that potential users of the data should keep in mind. First, concerning the M&A of ESG information firms, there might be some M&A events that could not be identified. This is because for four firms that are no longer active as of June 2023 (see comments column in sheet 4) it could not be determined whether the inactivity is the outcome of a cessation of activity or an M&A event. Second, with regards to the Headquarters variable (sheet 5), for 7 merged companies that no longer have any web presence no head office could be found. To avoid missing values, the headquarters of these companies were attributed to the capital of their country (2 times Paris, once respectively Canberra, Amsterdam, Madrid, London, and Stockholm). Also, as a partnership the firm McKinsey has varying head offices depending on who is the managing partner. For simplification, the New York office, one of the largest presences of the company was chosen as headquarters.

## Ethics Statement

The authors confirm that they have read the ethical requirements for publication in Data in Brief. Furthermore, the authors confirm that the data presented do not involve human subjects, animal experiments, or any data collected from social media platforms.

As the data contained in the dataset are already part of the public domain, no permission to use primary data was needed.

## CRediT authorship contribution statement

**Andreas Dimmelmeier:** Conceptualization, Data curation, Methodology, Visualization, Writing – review & editing.

## Data Availability

ESG Firms Dataset (ESGFiDa) (Original data) (Open Science Foundation OSF).

## Footnotes

1 This number includes the values from the edgelist on sheet 6.

2 With one exception of a report in French.
